# Examination of Adverse Reactions After COVID-19 Vaccination Among Patients With a History of Multisystem Inflammatory Syndrome in Children

**DOI:** 10.1001/jamanetworkopen.2022.48987

**Published:** 2023-01-03

**Authors:** Matthew D. Elias, Dongngan T. Truong, Matthew E. Oster, Felicia L. Trachtenberg, Xiangyu Mu, Pei-Ni Jone, Elizabeth C. Mitchell, Kirsten B. Dummer, S. Kristen Sexson Tejtel, Onyekachukwu Osakwe, Deepika Thacker, Jennifer A. Su, Tamara T. Bradford, Kristin M. Burns, M. Jay Campbell, Thomas J. Connors, Laura D’Addese, Daniel Forsha, Olivia H. Frosch, Therese M. Giglia, Lauren R. Goodell, Stephanie S. Handler, Keren Hasbani, Camden Hebson, Anita Krishnan, Sean M. Lang, Brian W. McCrindle, Kimberly E. McHugh, Lerraughn M. Morgan, R. Mark Payne, Arash Sabati, Eyal Sagiv, Yamuna Sanil, Faridis Serrano, Jane W. Newburger, Audrey Dionne

**Affiliations:** 1 Division of Cardiology, The Children’s Hospital of Philadelphia, Philadelphia, Pennsylvania; 2 Division of Pediatric Cardiology, University of Utah, Primary Children’s Hospital, Salt Lake City; 3 Children’s Healthcare of Atlanta, Emory University School of Medicine, Atlanta, Georgia; 4 HealthCore Inc, Newton, Massachusetts; 5 Department of Pediatrics, Pediatric Cardiology, Children’s Hospital Colorado, University of Colorado, Anschutz Medical Campus, Aurora; 6 Cohen Children’s Medical Center, Northwell Health, New Hyde Park, New York; 7 Division of Pediatric Cardiology, Department of Pediatrics, University of California, San Diego, School of Medicine and Rady Children’s Hospital, San Diego, California; 8 Baylor College of Medicine, Texas Children’s Hospital, Houston; 9 University of Mississippi Medical Center, Jackson; 10 Nemours Children’s Hospital, Wilmington, Delaware; 11 Division of Cardiology, Children’s Hospital Los Angeles, Los Angeles, California; 12 Children’s Hospital of New Orleans, New Orleans, Louisiana; 13 National Heart, Lung, and Blood Institute, National Institutes of Health, Bethesda, Maryland; 14 Division of Pediatric Cardiology, Department of Pediatrics, Duke University Medical Center, Durham, North Carolina; 15 Department of Pediatrics, Columbia University Vagelos College of Physicians and Surgeons and New York-Presbyterian Morgan Stanley Children’s Hospital, New York, New York; 16 The Heart Institute, Joe DiMaggio Children’s Hospital, Hollywood, Florida; 17 Ward Family Heart Center, Children’s Mercy Kansas City, Kansas City, Missouri; 18 Division of Pediatric Cardiology, C.S. Mott Children’s Hospital, University of Michigan, Ann Arbor; 19 Heart Center, Ann & Robert H. Lurie Children’s Hospital of Chicago, Chicago, Illinois; 20 Department of Pediatrics, Division of Pediatric Cardiology, Medical College of Wisconsin, Milwaukee; 21 Dell Children’s Medical Center, The University of Texas at Austin; 22 Department of Pediatrics, Division of Pediatric Cardiology, University of Alabama at Birmingham, Birmingham; 23 Division of Cardiology, Children’s National Hospital, Washington, DC; 24 The Heart Institute, Cincinnati Children’s Hospital Medical Center, Cincinnati, Ohio; 25 Department of Pediatrics, University of Toronto, Labatt Family Heart Centre, The Hospital for Sick Children, Toronto, Canada; 26 Department of Pediatrics, Medical University of South Carolina, Charleston; 27 Valley Children’s Healthcare and Hospital, Madera, California; 28 Riley Hospital for Children, Indiana University School of Medicine, Indianapolis; 29 Center for Heart Care, Phoenix Children’s Hospital, Phoenix, Arizona; 30 Division of Pediatric Cardiology, Seattle Children’s Hospital and the University of Washington School of Medicine, Seattle, Washington; 31 Division of Pediatric Cardiology, Department of Pediatrics, Children’s Hospital of Michigan, Central Michigan University, Detroit, Michigan; 32 Department of Cardiology, Boston Children’s Hospital, Boston, Massachusetts; 33 Department of Pediatrics, Harvard Medical School, Boston, Massachusetts

## Abstract

**Importance:**

Data are limited regarding adverse reactions after COVID-19 vaccination in patients with a history of multisystem inflammatory syndrome in children (MIS-C). The lack of vaccine safety data in this unique population may cause hesitancy and concern for many families and health care professionals.

**Objective:**

To describe adverse reactions following COVID-19 vaccination in patients with a history of MIS-C.

**Design, Setting, and Participants:**

In this multicenter cross-sectional study including 22 North American centers participating in a National Heart, Lung, and Blood Institute, National Institutes of Health–sponsored study, Long-Term Outcomes After the Multisystem Inflammatory Syndrome in Children (MUSIC), patients with a prior diagnosis of MIS-C who were eligible for COVID-19 vaccination (age ≥5 years; ≥90 days after MIS-C diagnosis) were surveyed between December 13, 2021, and February 18, 2022, regarding COVID-19 vaccination status and adverse reactions.

**Exposures:**

COVID-19 vaccination after MIS-C diagnosis.

**Main Outcomes and Measures:**

The main outcome was adverse reactions following COVID-19 vaccination. Comparisons were made using the Wilcoxon rank sum test for continuous variables and the χ^2^ or Fisher exact test for categorical variables.

**Results:**

Of 385 vaccine-eligible patients who were surveyed, 185 (48.1%) received at least 1 vaccine dose; 136 of the vaccinated patients (73.5%) were male, and the median age was 12.2 years (IQR, 9.5-14.7 years). Among vaccinated patients, 1 (0.5%) identified as American Indian/Alaska Native, non-Hispanic; 9 (4.9%) as Asian, non-Hispanic; 45 (24.3%) as Black, non-Hispanic; 59 (31.9%) as Hispanic or Latino; 53 (28.6%) as White, non-Hispanic; 2 (1.1%) as multiracial, non-Hispanic; and 2 (1.1%) as other, non-Hispanic; 14 (7.6%) had unknown or undeclared race and ethnicity. The median time from MIS-C diagnosis to first vaccine dose was 9.0 months (IQR, 5.1-11.9 months); 31 patients (16.8%) received 1 dose, 142 (76.8%) received 2 doses, and 12 (6.5%) received 3 doses. Almost all patients received the BNT162b2 vaccine (347 of 351 vaccine doses [98.9%]). Minor adverse reactions were observed in 90 patients (48.6%) and were most often arm soreness (62 patients [33.5%]) and/or fatigue (32 [17.3%]). In 32 patients (17.3%), adverse reactions were treated with medications, most commonly acetaminophen (21 patients [11.4%]) or ibuprofen (11 [5.9%]). Four patients (2.2%) sought medical evaluation, but none required testing or hospitalization. There were no patients with any serious adverse events, including myocarditis or recurrence of MIS-C.

**Conclusions and Relevance:**

In this cross-sectional study of patients with a history of MIS-C, no serious adverse events were reported after COVID-19 vaccination. These findings suggest that the safety profile of COVID-19 vaccination administered at least 90 days following MIS-C diagnosis appears to be similar to that in the general population.

## Key Points

**Question:**

Are patients with a history of multisystem inflammatory syndrome in children (MIS-C) at increased risk of adverse reactions from COVID-19 vaccination?

**Findings:**

In this multicenter cross-sectional study of 385 patients aged 5 years or older with prior MIS-C who were eligible for COVID-19 vaccination, 48% received at least 1 dose. Mild adverse reactions occurred in 49% (most often arm soreness [34%] and/or fatigue [17%]), and no patients reported any serious adverse events, including myocarditis or recurrence of MIS-C.

**Meaning:**

In this study, a history of MIS-C was not associated with an increased risk of serious adverse reactions following COVID-19 vaccination.

## Teaser

This cross-sectional study examines adverse reactions after COVID-19 vaccination among patients aged 5 years or older who have a history of multisystem inflammatory syndrome in children.

## Introduction

Multisystem inflammatory syndrome in children (MIS-C) is a rare, severe inflammatory response that occurs a few weeks after SARS-CoV-2 infection. COVID-19 vaccines have been shown to decrease the risk of severe COVID-19 infection and complications,^1,2,3^ but limited data exist regarding safety of vaccination after MIS-C. The current recommendation from the Centers for Disease Control and Prevention (CDC) is to delay COVID-19 vaccination until at least 90 days after MIS-C diagnosis.^4^ It is unclear, though, if patients are at risk of recurrence of the same dysregulated immune response following vaccination.

The lack of data on vaccination safety in patients with prior MIS-C may cause hesitancy and concern for some families and health care professionals. Thus, we aimed to describe the adverse reactions following COVID-19 vaccination in this population.

## Methods

This cross-sectional study followed the Strengthening the Reporting of Observational Studies in Epidemiology (STROBE) reporting guideline. Patients with a history of MIS-C who were enrolled in a National Heart, Lung, and Blood Institute, National Institutes of Health–sponsored multicenter prospective observational cohort study, Long-Term Outcomes After the Multisystem Inflammatory Syndrome in Children (MUSIC), were included.^5^ MUSIC’s eligibility and methods have previously been published.^5^ Each of the participating North American sites was invited to contact all vaccine-eligible participants from December 13, 2021, to February 18, 2022, to complete a questionnaire (eAppendix in Supplement 1) regarding COVID-19 vaccination status and adverse reactions. Vaccine eligibility was defined as age 5 years or older and at least 90 days since MIS-C diagnosis. Patients who received vaccine prior to being diagnosed with MIS-C were excluded. This study was approved by the institutional review board of each site as part of MUSIC. Written informed consent was obtained for all participants; patients enrolled in MUSIC who had a waiver of consent were excluded from this analysis.

### Statistical Analysis

Descriptive statistics were obtained for baseline characteristics, vaccination status, and adverse reactions and are provided as numbers with percentages for discrete variables and medians with IQRs for continuous variables. Data about race and ethnicity were obtained via electronic medical records or provided by the participants using the 5 categories for race standards from the CDC (American Indian/Alaska Native, non-Hispanic; Asian, non-Hispanic; Black, non-Hispanic; Native Hawaiian/Other Pacific Islander; and White, non-Hispanic); we also included the following categories: Hispanic or Latino; multiracial, non-Hispanic; other, non-Hispanic (“other” reported by the participant or in the medical records); and unknown or not provided. This information was pertinent to this study to examine whether there were any differences in vaccinations and adverse events among racial and ethnic groups. To compare baseline characteristics between nonvaccinated and vaccinated patients, the Wilcoxon rank sum test was used for continuous variables and the χ^2^ test for categorical variables. To compare vaccination adverse reaction data between the first and second dose, the Wilcoxon rank sum test was used for continuous variables and the Fisher exact test for categorical variables. Statistical testing by vaccine dose excluded the third dose due to insufficient sample size. Participants who received a COVID-19 vaccine dose less than 90 days after being diagnosed with MIS-C were excluded from analysis but reported separately. Data were analyzed using SAS Enterprise Guide, version 7.15 (SAS Institute Inc). Two-sided *P* < .05 was considered significant.

## Results

### Vaccination Status

Among 1207 patients enrolled in MUSIC, 895 (74.2%) were eligible for COVID-19 vaccination at the time of data collection. Among these patients, 385 (43.0%) were successfully contacted across 22 sites (Table 1 and eFigure in Supplement 1) and 185 patients (48.1%) received at least 1 vaccine dose. The majority of vaccinated patients were male (136 [73.5%] vs 49 [26.5%] female), and the median age at first vaccine dose was 12.2 years (IQR, 9.5-14.7 years); 87 patients (47.0%) were 5 to 11 years of age, 93 (50.3%) were 12 to 17 years of age, and 5 (2.7%) were 18 to 20 years of age. Of the vaccinated patients, 1 (0.5%) identified as American Indian/Alaska Native, non-Hispanic; 9 (4.9%) as Asian, non-Hispanic; 45 (24.3%) as Black, non-Hispanic; 59 (31.9%) as Hispanic or Latino; 53 (28.6%) as White, non-Hispanic; 2 (1.1%) as multiracial, non-Hispanic; and 2 (1.1%) as other, non-Hispanic; 14 (7.6%) had unknown or undeclared race and ethnicity. None identified as Native Hawaiian/Pacific Islander, non-Hispanic. The median time from MIS-C diagnosis to first vaccine dose was 9.0 months (IQR, 5.1-11.9 months); 31 patients (16.8%) received 1 dose; 142 (76.8%), 2 doses; and 12 (6.5%), 3 doses. Almost all patients received the BNT162b2 vaccine (347 of 351 vaccine doses [98.9%]).

**Table 1.** Demographic and Baseline Characteristics of Vaccine-Eligible Participants With and Without COVID-19 Vaccination Following MIS-C

Characteristic	Participants^a^		*P* value^b^	Vaccinated participants^a^		*P* value^b^^,^^c^
	Vaccinated (n = 185)	Not vaccinated (n = 200)		1 Vaccine dose (n = 31)	2 Vaccine doses (n = 142)	
Age, median (IQR), y						
At admission for MIS-C	11.5 (8.3-14.3)	9.6 (7.3-13.2)	.01	12.7 (7.3-14.7)	11 .0 (8.0-13.4)	.33
At first vaccine dose	12.2 (9.5-14.7)	NA	NA	13.1 (8.0-15.6)	11.7 (9.2-14.2)	.50
Sex						
Female	49 (26.5)	74 (37.0)	.03	11 (35.5)	37 (26.1)	.29
Male	136 (73.5)	126 (63.0)		20 (64.5)	105 (73.9)	
Race and ethnicity^d^						
American Indian/Alaska Native, non-Hispanic	1 (0.5)	0	.048	0	1 (0.7)	.08
Asian, non-Hispanic	9 (4.9)	2 (1.0)		5 (16.1)	4 (2.8)	
Black, non-Hispanic	45 (24.3)	42 (21.0)		7 (22.6)	36 (25.4)	
Hispanic or Latino	59 (31.9)	54 (27.0)		11 (35.5)	44 (31.0)	
White, non-Hispanic	53 (28.6)	87 (43.5)		8 (25.8)	41 (28.9)	
Multiracial, non-Hispanic	2 (1.1)	3 (1.5)		0	2 (1.4)	
Other, non-Hispanic^e^	2 (1.1)	1 (0.5)		0	2 (1.4)	
Unknown or not provided	14 (7.6)	11 (5.5)		0	12 (8.5)	
Eligible individuals in participant’s household who were vaccinated for COVID-19						
All	137 (74.1)	31 (15.5)	.001	15 (48.4)	110 (77.5)	.09
Some	24 (13.0)	65 (32.5)		6 (19.4)	18 (12.7)	
None	0	46 (23.0)		0	0	
Missing	24 (13.0)	58 (29.0)		10 (32.3)	14 (9.9)	
Time from MIS-C diagnosis to vaccine dose, median (IQR), mo	9.0 (5.1-11.9)	NA	NA	8.2 (4.4-12.3)	9.0 (5.4-11.9)	.45
Time from vaccine eligibility to data collection deadline, median (IQR), mo^f^	5.9 (3.5-9.3)	9.5 (5.1-10.5)	.001	5.9 (3.5-9.3)	3.5 (3.5-9.3)	.91
Hospitalization for MIS-C						
Length of stay, median (IQR), d	5 (4-8)	5 (4-7)	.29	7 (4-8)	5 (4-7)	.09
Intensive care unit admission, No./total No. (%)	90/145 (62.1)	94/144 (65.3)	.57	18/25 (72.0)	64/108 (59.3)	.24
Ventricular dysfunction, No./total No. (%)	67/145 (46.2)	69/147 (46.9)	.90	15/24 (62.5)	46/109 (42.2)	.07
Mechanical ventilation, No./total No. (%)	8/146 (5.5)	7/147 (4.8)	.59	2/24 (8.3)	4/110 (3.6)	.88
Vasoactive infusion, No./total No. (%)	68/146 (46.6)	71/147 (48.3)	.77	13/24 (54.2)	48/110 (43.6)	.35

Abbreviations: MIS-C, multisystem inflammatory syndrome in children; NA, not applicable.

^a^ Data are presented as number (percentage) of participants unless otherwise indicated. Numbers in the column headings represent the number of vaccine-eligible patients (age ≥5 years and ≥90 days after MIS-C diagnosis) as of the data collection deadline of February 18, 2022, and were used as denominators to calculate percentages of patients with data collected.

^b^ The Wilcoxon rank sum test was used for continuous variables and the χ^2^ test for categorical variables.

^c^ Statistical testing excluded the third vaccine dose due to insufficient sample size.

^d^ No patients identified as Native Hawaiian/Pacific Islander, non-Hispanic.

^e^ “Other” was provided for race and ethnicity by the participant or in the medical records.

^f^ Date of vaccination eligibility was determined by age (≥5 years), time since hospital admission for MIS-C (≥90 days), and approval of vaccination for use in the participant’s age group and country.

Compared with nonresponders, survey participants were older (median age, 10.3 years [IQR, 7.6-13.9 years] vs 9.8 years [IQR, 7.2-12.7 years]; *P* = .02) and more often male (262 of 385 [68.1%] vs 299 of 510 [58.6%]; *P* = .004). Hispanic or Latino patients and White, non-Hispanic patients were more likely to have vaccine data collected than Black, non-Hispanic patients, and survey participants more frequently had ventricular dysfunction during prior MIS-C hospitalization (136 of 292 [46.6%] vs 144 of 376 [38.3%]; *P* = .03). There were no significant differences in length of prior admission for MIS-C (median, 5 days [IQR, 4-7 days] vs 5 days [IQR, 4-8 days]; *P* = .65) or severity of prior MIS-C, including intensive care unit admission (184 of 289 [63.7%] vs 261 of 379 [68.9%]; *P* = .15), mechanical ventilation (15 of 293 [5.1%] vs 22 of 377 [5.8%]; *P* = .68), or vasoactive infusion (139 of 293 [47.4%] vs 174 of 376 [46.3%]; *P* = .76) (eTable 1 in Supplement 1).

All 185 vaccinated and 200 nonvaccinated patients were vaccine eligible, but vaccinated patients were older at the time of MIS-C diagnosis (median age, 11.5 years [IQR, 8.3-14.3 years] vs 9.6 years [IQR, 7.3-13.2 years]; *P* = .01), more often male (136 [73.5%] vs 126 [63.0%]; *P* = .03), and more likely to have all household members vaccinated (137 [74.1%] vs 31 [15.5%]; *P* = .001); they also had a shorter period since becoming vaccine eligible (median, 5.9 months [IQR, 3.5-9.3 months] vs 9.5 months [IQR, 5.1-10.5 months]; *P* = .001), and White, non-Hispanic patients were less likely to be vaccinated (Table 1 and eTable 2 in Supplement 1). There was no significant difference between vaccinated and nonvaccinated patients in length of hospital admission for MIS-C (median, 5 days [IQR, 4-8 days] vs 5 days [IQR, 4-7 days]; *P* = .29) or severity of prior MIS-C (intensive care unit admission: 90 of 145 [62.1%] vs 94 of 144 [65.3%]; *P* = .57; ventricular dysfunction: 67 of 145 [46.2%] vs 69 of 147 [46.9%]; *P* = .90; mechanical ventilation: 8 of 146 [5.5%] vs 7/147 [4.8%]; *P* = .59; vasoactive infusion: 68 of 146 [46.6%] vs 71 of 147 [48.3%]; *P* = .77).

### Adverse Reactions

Questionnaires were completed 1 to 304 days after vaccination (median, 81 days [IQR, 48-193 days] after dose 1; 63 days [IQR, 36-173 days] after dose 2; 30 days [IQR, 24-38 days] after dose 3). Of those vaccinated, 90 (48.6%) reported at least 1 adverse reaction, most commonly arm soreness (62 [33.5%]) and/or fatigue (32 [17.3%]). Fever occurred in 21 patients (11.4%), all within the first day of receiving the vaccine dose and lasting a median of 1 day (range, 1-5 days). Adverse reactions were treated with medication in 32 patients (17.3%), most often acetaminophen (21 patients [11.4%]) or ibuprofen (11 patients [5.9%]). There were no significant differences in adverse reactions between the first and second doses (Table 2) regardless of age or prior MIS-C severity (eTables 3 and 4 in Supplement 1). Four patients (2.2%) sought medical evaluation after the first or second dose, including a phone call to their primary care physician in 2 cases (1.1%), 1 outpatient visit with a primary care physician (0.5%), and 1 urgent care or emergency department evaluation (0.5%); no patient sought medical evaluation after the third dose (n = 12) (eTable 5 in Supplement 1). Symptoms leading to medical care included abdominal pain, conjunctival injection, fever, chills, fatigue, arm soreness, arm redness and/or swelling, and rash. Of the 4 patients who sought medical evaluation, the only medications administered included 1 patient (25.0%) receiving acetaminophen and 1 (25.0%) receiving ibuprofen. No patients required further testing, including chest x-rays, electrocardiography, echocardiography, heart rhythm monitoring, troponin level measurement, or cardiac magnetic resonance imaging, and no patients were hospitalized (Figure).

**Table 2.** Adverse Reactions Associated With COVID-19 Vaccination After MIS-C

	Patients, No. (%)^a^			*P* value^b^
	Any vaccine dose (N = 185)	First dose (n = 185)	Second dose (n = 154)	
Vaccine				
BNT162b2	NA	183 (98.9)	153 (99.4)	NA
mRNA-1273	NA	1 (0.5)	1 (0.6)	NA
Other or unknown	NA	1 (0.5)	0	NA
Time since last vaccine dose, median (IQR), d	NA	NA	21 (21-28)	NA
Adverse reaction				
Any	90 (48.6)	66 (35.7)	63 (40.9)	.14
Arm soreness	62 (33.5)	48 (25.9)	40 (26.0)	.25
Fatigue	32 (17.3)	26 (14.1)	18 (11.7)	.28
Fever	21 (11.4)	11 (5.9)	12 (7.8)	.59
Headache	14 (7.6)	8 (4.3)	10 (6.5)	.49
Myalgia	11 (5.9)	6 (3.2)	4 (2.6)	.50
Arm redness and/or swelling	8 (4.3)	5 (2.7)	5 (3.2)	.52
Chills	7 (3.8)	4 (2.2)	5 (3.2)	.56
Rash	7 (3.8)	4 (2.2)	4 (2.6)	.63
Abdominal pain	3 (1.6)	0	3 (1.9)	.13
Nausea or vomiting	2 (1.1)	1 (0.5)	1 (0.6)	.69
Palpitations	2 (1.1)	1 (0.5)	1 (0.6)	.69
Other^c^	16 (8.6)	11 (5.9)	11 (7.1)	.81
Medical evaluation among those with adverse reactions	4 (2.2)	2 (1.1)	2 (1.3)	.68
Medical tests performed due to adverse reactions	0	0	0	NA
Medication taken for adverse reactions	32 (17.3)	20 (10.8)	20 (13.0)	.73
Acetaminophen	21 (11.4)	12 (6.5)	16 (10.4)	.24
Ibuprofen	11 (5.9)	8 (4.3)	6 (3.9)	>.99
Other^d^	3 (1.6)	1 (0.5)	1 (0.6)	>.99

Abbreviations: MIS-C, multisystem inflammatory syndrome in children; NA, not applicable.

^a^ Numbers in the column headings represent the number of patients who received a vaccine and were used as denominators to calculate percentages of patients with data collected.

^b^ The Wilcoxon rank sum test was used for continuous variables. The Fisher exact test was used to compare the first and second dose.

^c^ Chest pain, diarrhea, and conjunctival injection were each reported once after the second dose, and lymphadenopathy was reported once each after the first and second dose. Other adverse reactions not specifically asked in the questionnaire but provided by patients included rhinorrhea, sore throat, dizziness, and hunger. Patients were asked about potential adverse reactions of shortness of breath and menstrual cycle changes, but none reported these reactions.

^d^ Other medications included topical diphenhydramine and bismuth subsalicylate. Patients were asked about antibiotics and immunomodulatory medications, but no patients reported receiving these medications.

**Figure.**
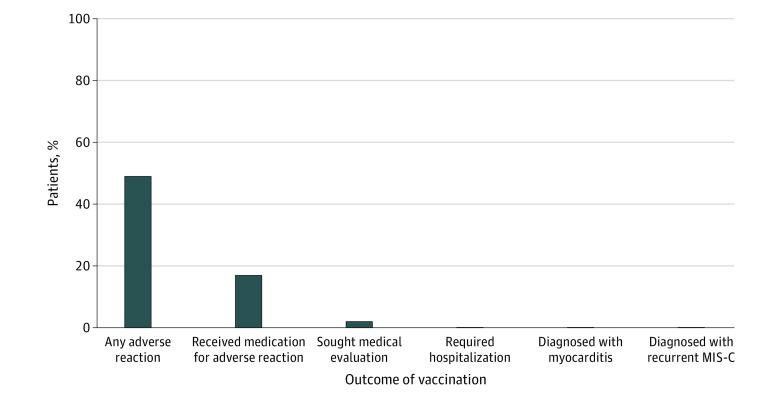
COVID-19 Vaccination Adverse Reaction Summary Among 185 Patients With a History of Multisystem Inflammatory Syndrome in Children (MIS-C)

The only episode of chest pain occurred in a female in the group aged 5 to 11 years 1 day after the second dose and lasted 1 day. She did not seek medical care or medication. Two patients (1.1%; males in the groups aged 12 to 17 years and 18 to 20 years) experienced palpitations 1 day after the first or second dose that lasted less than 48 hours; neither sought medical evaluation. No patients were evaluated for concerns of myocarditis or MIS-C recurrence.

Of note, 10 patients received at least 1 vaccine dose less than 90 days (range, 48-88 days) after MIS-C diagnosis, and 3 received 2 doses of vaccine. Two of these patients (20.0%) reported arm soreness, and 1 (10.0%) self-treated with ibuprofen. No serious adverse events were reported, and none of these patients sought medical evaluation.

## Discussion

Among respondents to this survey of COVID-19 vaccine–eligible patients with prior MIS-C, 48.1% received at least 1 vaccine dose, which is generally comparable with the national data; per the CDC, 39% of children aged 5 to 11 years and 71% of adolescents aged 12 to 17 years have received at least 1 vaccine dose.^6^ Among those who were vaccinated in this cohort with MIS-C, the patient-reported adverse reactions were mild, with no diagnosis of myocarditis or recurrent MIS-C. No patients with prior MIS-C required medical testing or hospitalization due to adverse reactions. The most common adverse events in the current study included arm soreness (33.5%), fatigue (17.3%), and fever (11.4%). Adverse reactions were similar to or less frequent than those in the general population; per the CDC, among individuals aged 5 to 11 years, 71% to 74% reported arm soreness, 34% to 39% reported fatigue, and 2% to 6% reported fever, and among individuals aged 12 to 15 years, 79% to 86% reported arm soreness, 60% to 66% reported fatigue, and 10% to 20% reported fever.^7^

Because MIS-C is associated with a dysregulated immune response to SARS-CoV-2,^8,9,10^ the possibility that a COVID-19 vaccine could trigger an abnormal inflammatory response has raised concerns.^11^ Small series of 15 and 32 patients reported no serious adverse events after vaccination.^12,13^ An international survey of health care professionals from 32 countries collected information from 273 vaccinated patients with prior MIS-C and reported no cases of MIS-C relapse.^14^ However, that study was limited by a lack of individual patient data. Our results are consistent with prior studies and add to the literature with detailed adverse reaction information, including patient interviews.

There is a rare risk of myocarditis after COVID-19 vaccination, especially after the second dose in adolescent males.^15,16^ To date, no cases of postvaccination myocarditis in patients with prior MIS-C have been reported. In this study, 3 patients experienced chest pain or palpitations, which resolved within 2 days. As these patients did not seek medical care, we could not determine whether there were laboratory or imaging markers of myocarditis; however, the mildness of the symptoms and rapid resolution suggest myocarditis was not present.

In the aforementioned international survey on COVID-19 vaccination after MIS-C, 20% of health care professionals considered MIS-C to be a contraindication for vaccination, and a significant number proposed longer intervals between MIS-C and vaccination or alternative vaccination schedules.^14^ Data from our study suggest that COVID-19 vaccination, when administered at least 90 days following MIS-C diagnosis, has a safety profile similar to that in the general population.

### Limitations

There are limitations to this study. Sites were unable to contact every vaccine-eligible patient, and some sites did not participate in the survey. There may be recall bias regarding mild symptoms, and families of nonvaccinated patients may have been less likely to return missed telephone calls. The rationale for receiving or not receiving vaccinations was also not obtained in this study, but available clinical and demographic data were obtained to compare vaccinated and nonvaccinated patients. Vaccination was reported by patients and families and could not be verified.

## Conclusions

In this cohort study of patients with a history of MIS-C, 48.1% of those surveyed were vaccinated for COVID-19 and none experienced serious adverse reactions, including a diagnosis of myocarditis or MIS-C recurrence. These findings support the CDC recommendation for COVID-19 vaccination at least 90 days following MIS-C diagnosis, with ongoing surveillance of adverse events.

## Notes

Supplement 1. **eFigure.** Participating MUSIC Sites **eTable 1.** Demographics and Baseline Characteristics of Vaccine-Eligible Participants With and Without Vaccination Data Collected **eTable 2.** Demographics and Baseline Characteristics of Vaccine Data Collected Among Participants With and Without COVID-19 Vaccination Following MIS-C, by Age Group **eTable 3.** COVID-19 Vaccination Adverse Reactions Following MIS-C, by Dose and Age Group **eTable 4.** COVID-19 Vaccination Adverse Reactions Following MIS-C, by MIS-C Severity **eTable 5.** Third COVID-19 Vaccination Dose Demographics, Baseline Characteristics, and Adverse Reactions **eAppendix.** Telephone Questionnaire for Vaccination Data Collection

Supplement 2.
Pediatric Heart Network MUSIC Study Investigators

Supplement 3.
Data Sharing Statement
