# Supplementary material for: Examination of Adverse Reactions After COVID-19 Vaccination Among Patients With a History of Multisystem Inflammatory Syndrome in Children
Source: JAMA Netw Open. 2023 Jan 3;6(1):e2248987. doi: 10.1001/jamanetworkopen.2022.48987 (PMC9857632; doi:10.1001/jamanetworkopen.2022.48987)
Supplement: Supplement 1. — eFigure. Participating MUSIC Sites eTable 1. Demographics and Baseline Characteristics of Vaccine-Eligible Participants With and Without Vaccination Data Collected eTable 2. Demographics and Baseline Characteristics of Vaccine Data Collected Among Participants With and Without COVID-19 Vaccination Following MIS-C, by Age Group eTable 3. COVID-19 Vaccination Adverse Reactions Following MIS-C, by Dose and Age Group eTable 4. COVID-19 Vaccination Adverse Reactions Following MIS-C, by MIS-C Severity eTable 5. Third COVID-19 Vaccination Dose Demographics, Baseline Characteristics, and Adverse Reactions eAppendix. Telephone Questionnaire for Vaccination Data Collection [file jamanetwopen-e2248987-s001.pdf]

# Supplementary Online Content

Elias MD, Truong DT, Oster ME, et al; for the Pediatric Heart Network MUSIC Study Investigators. Examination of adverse reactions after COVID-19 vaccination among patients with a history of multisystem inflammatory syndrome in children. *JAMA Netw Open*. 2023;6(1):e2248987. doi:10.1001/jamanetworkopen.2022.48987

**eFigure.** Participating MUSIC Sites

**eTable 1.** Demographics and Baseline Characteristics of Vaccine-Eligible Participants With and Without Vaccination Data Collected

**eTable 2.** Demographics and Baseline Characteristics of Vaccine Data Collected Among Participants With and Without COVID-19 Vaccination Following MIS-C, by Age Group

**eTable 3.** COVID-19 Vaccination Adverse Reactions Following MIS-C, by Dose and Age Group

**eTable 4.** COVID-19 Vaccination Adverse Reactions Following MIS-C, by MIS-C Severity

**eTable 5.** Third COVID-19 Vaccination Dose Demographics, Baseline Characteristics, and Adverse Reactions

**eAppendix.** Telephone Questionnaire for Vaccination Data Collection

This supplementary material has been provided by the authors to give readers additional information about their work.

© 2023 Elias MD et al. *JAMA Network Open*.

## eFigure: Participating MUSIC Sites

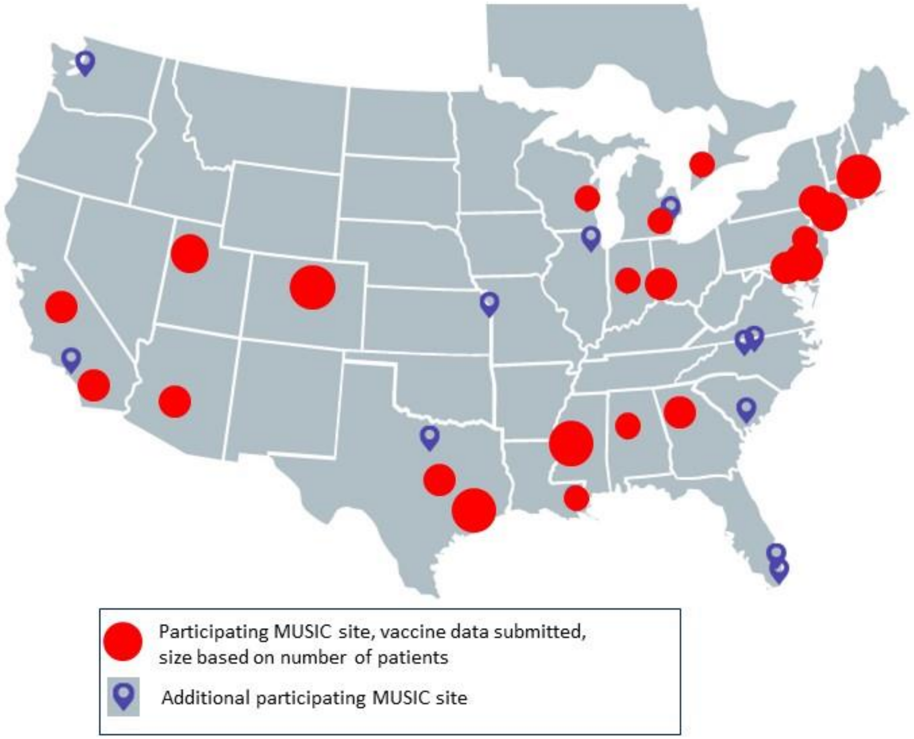


**Figure Legend:**

Sites that contributed vaccine data include: Boston Children's Hospital, MA; Children's Healthcare of Atlanta, GA; Children's Hospital Colorado, CO; Children's Hospital New Orleans, LA; Children's Hospital of Philadelphia, PA; Children's National Hospital, DC; Children's Wisconsin, WI; Cincinnati Children's Hospital Medical Center, OH; Cohen Children's Hospital, NY; Dell Medical Center, CA; Hospital for Sick Children, Toronto ON; Morgan Stanley Children's Hospital, NY; Nemours Children's Hospital, DE; Phoenix Children Hospital, AZ; Primary Children's Hospital, UT; Rady Children's Hospital, CA; Riley Children's Hospital, IN; Texas Children's Hospital, TX; University of Alabama, AL; University of Michigan Health System, MI; University of Mississippi, MS; Valley Children's Healthcare and Hospital, CA.

© 2023 Elias MD et al. *JAMA Network Open*.

**eTable 1: Demographics and Baseline Characteristics of Vaccine-Eligible Participants With and Without Vaccination Data Collected**

| | Vaccine-eligible with data collected N=385 | Vaccine-eligible without data collected N=510 | P value[a] |
|---|---|---|---|
| **Age at time of MIS-C admission**, years (median, IQR) | 10.3 (7.6-13.9) | 9.8 (7.2-12.7) | 0.02 |
| **Sex** | | | 0.004 |
| Male, n (%) | 262 (68.1) | 299 (58.6) | |
| Female, n (%) | 123 (31.9) | 211 (41.4) | |
| **Race/Ethnicity** | | | 0.007 |
| American Indian/Alaska Native, non-Hispanic, n (%) | 1 (0.3) | 0 (0.0) | |
| Asian, non-Hispanic, n (%) | 11 (2.9) | 21 (4.1) | |
| Black, non-Hispanic, n (%) | 87 (22.6) | 153 (30.0) | |
| Hawaiian Native/Pacific Islander, non-Hispanic, n (%) | 0 (0.0) | 1 (0.2) | |
| Hispanic or Latino, n (%) | 113 (29.4) | 121 (23.7) | |
| White, non-Hispanic, n (%) | 140 (36.4) | 144 (28.2) | |
| Multi-Racial, non-Hispanic, n (%) | 5 (1.3) | 18 (3.5) | |
| Other, non-Hispanic, n (%) | 3 (0.8) | 6 (1.2) | |
| Unknown or Refused, n (%) | 25 (6.5) | 46 (9.0) | |
| **MIS-C hospitalization** | | | |
| Length of stay, days (median, IQR) | 5 (4-7) | 5 (4-8) | 0.65 |

© 2023 Elias MD et al. *JAMA Network Open*.

| | Vaccine-eligible with data collected<br><br>N=385 | Vaccine-eligible without data collected<br><br>N=510 | P value[a] |
|---|---|---|---|
| Intensive care unit admission, n/N (%) | 184/289 (63.7) | 261/379 (68.9) | 0.15 |
| Ventricular dysfunction, n/N (%) | 136/292 (46.6) | 144/376 (38.3) | 0.03 |
| Mechanical ventilation, n/N (%) | 15/293 (5.1) | 22/377 (5.8) | 0.68 |
| Vasoactive infusions, n/N (%) | 139/293 (47.4) | 174/376 (46.3) | 0.76 |

[a] Wilcoxon rank sum test for continuous variables and Chi-Square test for categorical variables

© 2023 Elias MD et al. *JAMA Network Open*.

## eTable 2: Demographics and Baseline Characteristics of Vaccine Data Collected Among Participants With and Without COVID-19 Vaccination Following MIS-C, by Age Group

| | No vaccine | | | ≥1 vaccine dose | | |
|---|---|---|---|---|---|---|
| | **5 to <12 years N=122** | **12 to <18 years N=67** | **18 to <21 years N=8** | **5 to <12 years N=87** | **12 to <18 years N=93** | **18 to <21 years N=5** |
| **Age at time of MIS-C admission**, years (median, IQR) | 7.8 (6.0-9.2) | 14.1 (12.5-15.2) | 18.0 (17.5-18.9) | 8.0 (6.1-9.7) | 13.8 (12.7-14.9) | 18.1 (18.0-19.4) |
| **Sex** | | | | | | |
| Male, n (%) | 73 (59.8) | 47 (70.1) | 5 (62.5) | 59 (67.8) | 72 (77.4) | 5 (100.0) |
| Female, n (%) | 49 (40.2) | 20 (29.9) | 3 (37.5) | 28 (32.2) | 21 (22.6) | 0 (0.0) |
| **Race/Ethnicity** | | | | | | |
| American Indian/Alaska Native, non-Hispanic, n (%) | 0 (0.0) | 0 (0.0) | 0 (0.0) | 0 (0.0) | 0 (0.0) | 1 (20.0) |
| Asian, non-Hispanic, n (%) | 2 (1.6) | 0 (0.0) | 0 (0.0) | 4 (4.6) | 4 (4.3) | 1 (20.0) |
| Black, non-Hispanic, n (%) | 17 (13.9) | 19 (28.4) | 4 (50.0) | 22 (25.3) | 23 (24.7) | 0 (0.0) |
| Hawaiian Native/Pacific Islander, non-Hispanic, n (%) | 0 (0.0) | 0 (0.0) | 0 (0.0) | 0 (0.0) | 0 (0.0) | 0 (0.0) |
| Hispanic or Latino, n (%) | 37 (30.3) | 16 (23.9) | 1 (12.5) | 26 (29.9) | 32 (34.4) | 1 (20.0) |
| White, non-Hispanic, n (%) | 53 (43.4) | 31 (46.3) | 3 (37.5) | 27 (31.0) | 26 (28.0) | 0 (0.0) |
| Multi-Racial, non-Hispanic, n (%) | 3 (2.5) | 0 (0.0) | 0 (0.0) | 1 (1.1) | 1 (1.1) | 0 (0.0) |
| Other, non-Hispanic, n (%) | 1 (0.8) | 0 (0.0) | 0 (0.0) | 1 (1.1) | 1 (1.1) | 0 (0.0) |
| Unknown or Refused, n (%) | 9 (7.4) | 1 (1.5) | 0 (0.0) | 6 (6.9) | 6 (6.5) | 2 (40.0) |

© 2023 Elias MD et al. *JAMA Network Open*.

| | No vaccine | | | ≥1 vaccine dose | | |
|---|---|---|---|---|---|---|
| | **5 to <12 years N=122** | **12 to <18 years N=67** | **18 to <21 years N=8** | **5 to <12 years N=87** | **12 to <18 years N=93** | **18 to <21 years N=5** |
| **Number of eligible individuals in participant's household who are vaccinated for COVID-19** | | | | | | |
| All, n (%) | 23 (18.9) | 7 (10.4) | 1 (12.5) | 72 (82.8) | 62 (66.7) | 3 (60.0) |
| Some, n (%) | 43 (35.2) | 19 (28.4) | 3 (37.5) | 9 (10.3) | 14 (15.1) | 1 (20.0) |
| None, n (%) | 32 (26.2) | 11 (16.4) | 3 (37.5) | 0 (0.0) | 0 (0.0) | 0 (0.0) |
| Missing, n (%) | 24 (19.7) | 30 (44.8) | 1 (12.5) | 6 (6.9) | 17 (18.3) | 1 (20.0) |
| **Time since MIS-C diagnosis**, months (median, IQR) | n/a | n/a | n/a | 9.6 (8.5-12.3) | 7.3 (4.9-11.3) | 5.8 (4.6-9.9) |
| **Time since vaccine eligibility to data collection deadline in February 2022**, months (median, IQR) | 9.5 (3.5-10.7) | 9.4 (5.1-10.3) | 9.6 (8.0-10.5) | 3.5 (3.5-3.5) | 9.3 (9.1-9.3) | 10.9 (10.8-11.1) |
| **MIS-C hospitalization** | | | | | | |
| Length of stay, days (median, IQR) | 5 (3-7) | 5 (4-7) | 10.5 (8-14) | 5 (4-7) | 6 (4-8) | 8 (6-10) |
| Intensive care admission, n (%) | 54/89 (60.7) | 33/47 (70.2) | 5/6 (83.3) | 36/65 (55.4) | 51/75 (68.0) | 3/5 (60.0) |
| Ventricular dysfunction, n (%) | 39/90 (43.3) | 26/49 (53.1) | 4/6 (66.7) | 28/65 (43.1) | 36/75 (48.0) | 3/5 (60.0) |
| Mechanical ventilation, n (%) | 6/90 (6.7) | 1/49 (2.0) | 0/6 (0.0) | 4/65 (6.2) | 4/76 (5.3) | 0/5 (0.0) |
| Vasoactive infusions, n (%) | 40/90 (44.4) | 26/49 (53.1) | 5/6 (83.3) | 31/65 (47.7) | 34/76 (44.7) | 3/5 (60.0) |

Age groups were determined based on age at first vaccination (for those with vaccination date) or age at data collection (for those without vaccination date).

© 2023 Elias MD et al. *JAMA Network Open*.

## eTable 3: COVID-19 Vaccination Adverse Reactions Following MIS-C, by Dose and Age Group

| | 5 to <12 years N=87 | | | 12 to <18 years N=93 | | | 18 to <21 years N=5 | | |
|---|---|---|---|---|---|---|---|---|---|
| | 1st dose N=87 | 2nd dose N=73 | 3rd dose N=0 | 1st dose N=93 | 2nd dose N=78 | 3rd dose N=11 | 1st dose N=5 | 2nd dose N=3 | 3rd dose N=1 |
| **Vaccination brand/manufacturer, n (%)** | | | | | | | | | |
| Pfizer, n (%) | 86 (98.9) | 73 (100.0) | 0 (0.0) | 93 (100.0) | 78 (100.0) | 11 (100.0) | 4 (80.0) | 2 (66.7) | 0 (0.0) |
| Moderna, n (%) | 0 (0.0) | 0 (0.0) | 0 (0.0) | 0 (0.0) | 0 (0.0) | 0 (0.0) | 1 (20.0) | 1 (33.3) | 1 (100.0) |
| Other or Unknown, n (%) | 1 (1.1) | 0 (0.0) | 0 (0.0) | 0 (0.0) | 0 (0.0) | 0 (0.0) | 0 (0.0) | 0 (0.0) | 0 (0.0) |
| **Number of patients who experienced any adverse reactions, n (%)** | 30 (34.5) | 32 (43.8) | 0 (0.0) | 32 (34.4) | 29 (37.2) | 6 (54.5) | 4 (80.0) | 2 (66.7) | 1 (100.0) |
| Arm soreness, n (%) | 22 (25.3) | 22 (30.1) | 0 (0.0) | 22 (23.7) | 16 (20.5) | 4 (36.4) | 4 (80.0) | 2 (66.7) | 1 (100.0) |
| Fatigue, n (%) | 7 (8.0) | 4 (5.5) | 0 (0.0) | 17 (18.3) | 14 (17.9) | 5 (45.5) | 2 (40.0) | 0 (0.0) | 1 (100.0) |
| Fever, n (%) | 6 (6.9) | 7 (9.6) | 0 (0.0) | 5 (5.4) | 5 (6.4) | 2 (18.2) | 0 (0.0) | 0 (0.0) | 0 (0.0) |
| Headache, n (%) | 3 (3.4) | 4 (5.5) | 0 (0.0) | 4 (4.3) | 6 (7.7) | 1 (9.1) | 1 (20.0) | 0 (0.0) | 0 (0.0) |
| Myalgia, n (%) | 4 (4.6) | 1 (1.4) | 0 (0.0) | 2 (2.2) | 3 (3.8) | 3 (27.3) | 0 (0.0) | 0 (0.0) | 0 (0.0) |
| Arm redness/swelling, n (%) | 3 (3.4) | 5 (6.8) | 0 (0.0) | 2 (2.2) | 0 (0.0) | 1 (9.1) | 0 (0.0) | 0 (0.0) | 0 (0.0) |
| Chills, n (%) | 0 (0.0) | 1 (1.4) | 0 (0.0) | 2 (2.2) | 3 (3.8) | 0 (0.0) | 2 (40.0) | 1 (33.3) | 0 (0.0) |
| Rash, n (%) | 2 (2.3) | 4 (5.5) | 0 (0.0) | 2 (2.2) | 0 (0.0) | 1 (9.1) | 0 (0.0) | 0 (0.0) | 0 (0.0) |
| Abdominal pain, n (%) | 0 (0.0) | 1 (1.4) | 0 (0.0) | 0 (0.0) | 2 (2.6) | 0 (0.0) | 0 (0.0) | 0 (0.0) | 0 (0.0) |
| Nausea or vomiting, n (%) | 0 (0.0) | 0 (0.0) | 0 (0.0) | 0 (0.0) | 1 (1.3) | 0 (0.0) | 1 (20.0) | 0 (0.0) | 0 (0.0) |
| Palpitations, n (%) | 0 (0.0) | 0 (0.0) | 0 (0.0) | 0 (0.0) | 1 (1.3) | 0 (0.0) | 1 (20.0) | 0 (0.0) | 0 (0.0) |
| Chest Pain, n (%) | 0 (0.0) | 1 (1.4) | 0 (0.0) | 0 (0.0) | 0 (0.0) | 0 (0.0) | 0 (0.0) | 0 (0.0) | 0 (0.0) |
| Diarrhea, n (%) | 0 (0.0) | 0 (0.0) | 0 (0.0) | 0 (0.0) | 1 (1.3) | 0 (0.0) | 0 (0.0) | 0 (0.0) | 0 (0.0) |
| Conjunctival injection, n (%) | 0 (0.0) | 1 (1.4) | 0 (0.0) | 0 (0.0) | 0 (0.0) | 0 (0.0) | 0 (0.0) | 0 (0.0) | 0 (0.0) |
| Lymphadenopathy, n (%) | 0 (0.0) | 0 (0.0) | 0 (0.0) | 1 (1.1) | 1 (1.3) | 0 (0.0) | 0 (0.0) | 0 (0.0) | 0 (0.0) |

© 2023 Elias MD et al. *JAMA Network Open*.

| | 5 to <12 years N=87 | | | 12 to <18 years N=93 | | | 18 to <21 years N=5 | | |
|---|---|---|---|---|---|---|---|---|---|
| | **1st dose N=87** | **2nd dose N=73** | **3rd dose N=0** | **1st dose N=93** | **2nd dose N=78** | **3rd dose N=11** | **1st dose N=5** | **2nd dose N=3** | **3rd dose N=1** |
| Shortness of breath, n (%) | 0 (0.0) | 0 (0.0) | 0 (0.0) | 0 (0.0) | 0 (0.0) | 0 (0.0) | 0 (0.0) | 0 (0.0) | 0 (0.0) |
| Menstrual cycle changes, n (%) | 0 (0.0) | 0 (0.0) | 0 (0.0) | 0 (0.0) | 0 (0.0) | 0 (0.0) | 0 (0.0) | 0 (0.0) | 0 (0.0) |
| Other adverse reactions, n (%) | 5 (5.7) | 5 (6.8) | 0 (0.0) | 5 (5.4) | 2 (2.6) | 2 (18.2) | 0 (0.0) | 0 (0.0) | 0 (0.0) |
| **Medical evaluation among those with adverse reactions, n (%)** | 0 (0.0) | 2 (2.7) | 0 (0.0) | 2 (2.2) | 0 (0.0) | 0 (0.0) | 0 (0.0) | 0 (0.0) | 0 (0.0) |
| **Medical tests performed due to adverse reactions, n (%)** | 0 (0.0) | 0 (0.0) | 0 (0.0) | 0 (0.0) | 0 (0.0) | 0 (0.0) | 0 (0.0) | 0 (0.0) | 0 (0.0) |
| **Medications due to adverse reactions, n (%)** | 9 (10.3) | 10 (13.7) | 0 (0.0) | 10 (10.8) | 10 (12.8) | 3 (27.3) | 1 (20.0) | 0 (0.0) | 0 (0.0) |
| Acetaminophen, n (%) | 5 (5.7) | 8 (11.0) | 0 (0.0) | 7 (7.5) | 8 (10.3) | 1 (9.1) | 0 (0.0) | 0 (0.0) | 0 (0.0) |
| Ibuprofen, n (%) | 5 (5.7) | 4 (5.5) | 0 (0.0) | 2 (2.2) | 2 (2.6) | 1 (9.1) | 1 (20.0) | 0 (0.0) | 0 (0.0) |
| Antibiotics, n (%) | 0 (0.0) | 0 (0.0) | 0 (0.0) | 0 (0.0) | 0 (0.0) | 0 (0.0) | 0 (0.0) | 0 (0.0) | 0 (0.0) |
| Other immunomodulatory medication, n (%) | 0 (0.0) | 0 (0.0) | 0 (0.0) | 0 (0.0) | 0 (0.0) | 0 (0.0) | 0 (0.0) | 0 (0.0) | 0 (0.0) |
| Other medications, n (%) | 0 (0.0) | 0 (0.0) | 0 (0.0) | 1 (1.1) | 1 (1.3) | 1 (9.1) | 0 (0.0) | 0 (0.0) | 0 (0.0) |

Note: N in the header represents the number of subjects who received vaccine and is used as the denominator for calculation of percentage with data collected.

© 2023 Elias MD et al. *JAMA Network Open*.

## eTable 4: COVID-19 Vaccination Adverse Reactions Following MIS-C, by MIS-C Severity

| | Vaccinated participants with severe MIS-C[a] N=110 | | | Vaccinated participants with non-severe MIS-C[a] N=75 | | |
|---|---|---|---|---|---|---|
| | 1st dose N=110 | 2nd dose N=88 | 3rd dose N=10 | 1st dose N=75 | 2nd dose N=66 | 3rd dose N=2 |
| **Vaccination brand/manufacturer, n (%)** | | | | | | |
| Pfizer, n (%) | 108 (98.2) | 87 (98.9) | 9 (90.0) | 75 (100.0) | 66 (100.0) | 2 (100.0) |
| Moderna, n (%) | 1 (0.9) | 1 (1.1) | 1 (10.0) | 0 (0.0) | 0 (0.0) | 0 (0.0) |
| Other or Unknown, n (%) | 1 (0.9) | 0 (0.0) | 0 (0.0) | 0 (0.0) | 0 (0.0) | 0 (0.0) |
| **Number of patients who experienced any adverse reactions, n (%)** | 35 (31.8) | 33 (37.5) | 60 (60.0) | 31 (41.3) | 30 (45.5) | 1 (50.0) |
| Arm soreness, n (%) | 24 (21.8) | 19 (21.6) | 4 (40.0) | 24 (32.0) | 21 (31.8) | 1 (50.0) |
| Fatigue, n (%) | 17 (15.5) | 14 (15.9) | 5 (50.0) | 9 (12.0) | 4 (6.1) | 1 (50.0) |
| Fever, n (%) | 7 (6.4) | 6 (6.8) | 1 (10.0) | 4 (5.3) | 6 (9.1) | 1 (50.0) |
| Headache, n (%) | 5 (4.5) | 6 (6.8) | 1 (10.0) | 3 (4.0) | 4 (6.1) | 0 (0.0) |
| Myalgia, n (%) | 4 (3.6) | 3 (3.4) | 2 (20.0) | 2 (2.7) | 1 (1.5) | 1 (50.0) |
| Arm redness/swelling, n (%) | 2 (1.8) | 1 (1.1) | 1 (10.0) | 3 (4.0) | 4 (6.1) | 0 (0.0) |
| Chills, n (%) | 3 (2.7) | 3 (3.4) | 0 (0.0) | 1 (1.3) | 2 (3.0) | 0 (0.0) |
| Rash, n (%) | 1 (0.9) | 1 (1.1) | 1 (10.0) | 3 (4.0) | 3 (4.5) | 0 (0.0) |
| Abdominal pain, n (%) | 0 (0.0) | 2 (2.3) | 0 (0.0) | 0 (0.0) | 1 (1.5) | 0 (0.0) |
| Nausea or vomiting, n (%) | 1 (0.9) | 1 (1.1) | 0 (0.0) | 0 (0.0) | 0 (0.0) | 0 (0.0) |
| Palpitations, n (%) | 1 (0.9) | 1 (1.1) | 0 (0.0) | 0 (0.0) | 0 (0.0) | 0 (0.0) |
| Chest Pain, n (%) | 0 (0.0) | 0 (0.0) | 0 (0.0) | 0 (0.0) | 1 (1.5) | 0 (0.0) |
| Diarrhea, n (%) | 0 (0.0) | 0 (0.0) | 0 (0.0) | 0 (0.0) | 1 (1.5) | 0 (0.0) |
| Conjunctival injection, n (%) | 0 (0.0) | 1 (1.1) | 0 (0.0) | 0 (0.0) | 0 (0.0) | 0 (0.0) |
| Lymphadenopathy, n (%) | 0 (0.0) | 0 (0.0) | 0 (0.0) | 1 (1.3) | 1 (1.5) | 0 (0.0) |

© 2023 Elias MD et al. *JAMA Network Open*.

| | Vaccinated participants with severe MIS-C[a] N=110 | | | Vaccinated participants with non-severe MIS-C[a] N=75 | | |
|---|---|---|---|---|---|---|
| | **1st dose N=110** | **2nd dose N=88** | **3rd dose N=10** | **1st dose N=75** | **2nd dose N=66** | **3rd dose N=2** |
| Shortness of breath, n (%) | 0 (0.0) | 0 (0.0) | 0 (0.0) | 0 (0.0) | 0 (0.0) | 0 (0.0) |
| Menstrual cycle changes, n (%) | 0 (0.0) | 0 (0.0) | 0 (0.0) | 0 (0.0) | 0 (0.0) | 0 (0.0) |
| Other adverse reactions, n (%) | 7 (6.4) | 4 (4.5) | 2 (20.0) | 3 (4.0) | 3 (4.5) | 0 (0.0) |
| **Medical evaluation among those with adverse reactions, n (%)** | 1 (0.9) | 2 (2.3) | 0 (0.0) | 1 (1.3) | 0 (0.0) | 0 (0.0) |
| **Medical tests performed due to adverse reactions, n (%)** | 0 (0.0) | 0 (0.0) | 0 (0.0) | 0 (0.0) | 0 (0.0) | 0 (0.0) |
| **Medications due to adverse reactions, n (%)** | 11 (10.0) | 12 (13.6) | 2 (20.0) | 9 (12.0) | 8 (12.1) | 1 (50.0) |
| Acetaminophen, n (%) | 7 (6.4) | 12 (13.6) | 1 (10.0) | 5 (6.7) | 4 (6.1) | 0 (0.0) |
| Ibuprofen, n (%) | 4 (3.6) | 2 (2.3) | 0 (0.0) | 4 (5.3) | 4 (6.1) | 1 (50.0) |
| Antibiotics, n (%) | 0 (0.0) | 0 (0.0) | 0 (0.0) | 0 (0.0) | 0 (0.0) | 0 (0.0) |
| Other immunomodulatory medication, n (%) | 0 (0.0) | 0 (0.0) | 0 (0.0) | 0 (0.0) | 0 (0.0) | 0 (0.0) |
| Other medications, n (%) | 1 (0.9) | 1 (1.1) | 1 (10.0) | 0 (0.0) | 0 (0.0) | 0 (0.0) |

[a] Severe MIS-C defined as ICU admission, ventricular dysfunction, mechanical ventilation, or vasoactive infusions.

© 2023 Elias MD et al. *JAMA Network Open*.

**eTable 5: Third COVID-19 Vaccination Dose Demographics, Baseline Characteristics, and Adverse Reactions**

| | **3 vaccine doses N=12** |
|---|---|
| **Age at time of MIS-C admission**, years (median, IQR) | 14.7 (13.4-15.9) |
| **Age at time of first vaccine dose**, years (median, IQR) | 15.3 (12.7-16.0) |
| **Sex** | |
| Male, n (%) | 11 (91.7) |
| Female, n (%) | 1 (8.3) |
| **Race/Ethnicity** | |
| Black, non-Hispanic, n (%) | 2 (16.7) |
| Hispanic or Latino, n (%) | 4 (33.3) |
| White, non-Hispanic, n (%) | 4 (33.3) |
| Unknown or Refused, n (%) | 2 (16.7) |
| **Number of eligible individuals in participant's household who are vaccinated for COVID-19** | |
| All, n (%) | 12 (100.0) |
| Some, n (%) | 0 (0.0) |
| None, n (%) | 0 (0.0) |
| Missing, n (%) | 0 (0.0) |
| **Time since MIS-C diagnosis**, months (median, IQR) | 9.5 (5.2-12.8) |
| **Time since vaccine eligibility to data collection deadline in February 2022**, months (median, IQR) | 9.3 (9.3-12.4) |

© 2023 Elias MD et al. *JAMA Network Open*.

| | 3 vaccine doses<br>N=12 |
|---|---|
| **MIS-C hospitalization** | |
| Length of stay, days (median, IQR) | 6.5 (4-9.5) |
| Intensive care unit admission, n/N (%) | 8/12 (66.7) |
| Ventricular dysfunction, n/N (%) | 6/12 (50.0) |
| Mechanical ventilation, n/N (%) | 2/12 (16.7) |
| Vasoactive infusions, n/N (%) | 7/12 (58.3) |
| **Vaccination brand/manufacturer** | |
| Pfizer-BioNTech, n (%) | 11 (91.7) |
| Moderna, n (%) | 1 (8.3) |
| **Time since last vaccine dose**, days (median, IQR) | 199 (177.5-215.5) |
| **Number of patients who experienced any adverse reactions, n (%)** | 7 (58.3) |
| Fatigue, n (%) | 6 (50.0) |
| Arm soreness, n (%) | 5 (41.7) |
| Myalgia, n (%) | 3 (25.0) |
| Fever, n (%) | 2 (16.7) |
| Headache, n (%) | 1 (8.3) |
| Arm redness/swelling, n (%) | 1 (8.3) |
| Rash, n (%) | 1 (8.3) |
| Other adverse reactions, n (%)[a] | 2 (16.7) |
| **Medical evaluation among those with adverse reactions, n (%)** | 0 (0.0) |

© 2023 Elias MD et al. *JAMA Network Open*.

| | 3 vaccine doses N=12 |
|---|---|
| **Medical tests performed due to adverse reactions, n (%)** | 0 (0.0) |
| **Medications due to adverse reactions, n (%)** | 3 (25.0) |
| Acetaminophen, n (%) | 1 (8.3) |
| Ibuprofen, n (%) | 1 (8.3) |
| Antibiotics, n (%) | 0 (0.0) |
| Other immunomodulatory medication, n (%) | 0 (0.0) |
| Other medications, n (%)[b] | 1 (8.3) |

Note: N in the header represents the number of vaccine-eligible subjects (age≥5 years and ≥90 days after MIS-C hospital admission) as of the data collection deadline of February 18, 2022 and is used as the denominator for calculation of percentage with data collected.
There were no patients with race identified as American Indian/Alaska Native, non-Hispanic; Asian, non-Hispanic; Hawaiian Native/Pacific Islander, non-Hispanic; Multi-Racial, non-Hispanic, or Other, non-Hispanic.
No patients reported chills, abdominal pain, nausea/vomiting, palpitations, chest pain, diarrhea, conjunctival injection, lymphadenopathy, shortness of breath, or menstrual cycle changes.
[a] Other adverse reactions included rhinorrhea, sore throat, dizziness and/or hunger.
[b] Other medications included topical diphenhydramine and/or bismuth subsalicylate.

© 2023 Elias MD et al. *JAMA Network Open*.

## eAppendix: Telephone Questionnaire for Vaccination Data Collection

| | | | |
|---|---|---|---|
| 1. | Subject ID | | ________________________ |
| 2. | Date of Data Collection (MM/DD/YYYY) | | ________________________ |
| 3. | Which COVID-19 vaccine dose does this form apply to? | | ☐ = First Dose<br>☐ = Second Dose<br>☐ = Third Dose<br>☐ = Fourth Dose |
| 4. | Did the participant receive this dose? | | ☐ Yes ☐ No *If no, skip to 10* |
| 5. | Is exact date of this COVID-19 dose known? | | ☐ Yes *(go to 5a)* ☐ No *(go to 5b)* |
| | 5a. | Please specify the date of the COVID-19 vaccine dose (MM/DD/YYYY) | ________________________ |
| | 5b. | Please provide the best estimate of the date of COVID-19 vaccination dose (MM/DD/YYYY) | ________________________ |
| 6. | Vaccination brand/manufacturer for this dose | | ☐ = Pfizer<br>☐ = Moderna<br>☐ = Johnson & Johnson<br>☐ = Novavax<br>☐ = Unknown<br>☐ = Other (specify): ____________ |

© 2023 Elias MD et al. *JAMA Network Open*.

| | | | |
|---|---|---|---|
| 7. | Did the participant experience any side effects after the vaccine dose? | | |
| | 7a. | Fever? | ☐ Yes ☐ No *If no, skip to 7b.* |
| | 7a1. | How many days after vaccination did symptom start? (Please write the approximate number of days) | ________________ |
| | 7a2. | How many days did the symptom last? (Please write the approximate number of days) | ________________ |
| | 7b. | Chest pains? | ☐ Yes ☐ No *If no, skip to 7c.* |
| | 7b1 | How many days after vaccination did symptom start? (Please write the approximate number of days) | ________________ |
| | 7b2. | How many days did the symptom last? (Please write the approximate number of days) | ________________ |
| | 7c. | Palpitations? | ☐ Yes ☐ No *If no, skip to 7d.* |
| | 7c1. | How many days after vaccination did symptom start? (Please write the approximate number of days) | ________________ |
| | 7c2. | How many days did the symptom last? (Please write the approximate number of days) | ________________ |
| | 7d. | Shortness of breath? | ☐ Yes ☐ No *If no, skip to 7e.* |
| | 7d1. | How many days after vaccination did symptom start? (Please write the approximate number of days) | ________________ |
| | 7d2. | How many days did the symptom last? (Please write the approximate number of days) | ________________ |
| | 7e. | Chills? | ☐ Yes ☐ No |
| | 7f. | Rash? | ☐ Yes ☐ No |
| | 7g. | Abdominal pain? | ☐ Yes ☐ No |
| | 7h. | Nausea or vomiting? | ☐ Yes ☐ No |

© 2023 Elias MD et al. *JAMA Network Open*.

| | | | |
|---|---|---|---|
| | 7i. | Diarrhea? | ☐ Yes ☐ No |
| | 7j. | Red eyes? | ☐ Yes ☐ No |
| | 7k. | Fatigue? | ☐ Yes ☐ No |
| | 7l. | Headache? | ☐ Yes ☐ No |
| | 7m. | Generalized muscle ache? | ☐ Yes ☐ No |
| | 7n. | Arm soreness? | ☐ Yes ☐ No |
| | 7o. | Are redness/swelling? | ☐ Yes ☐ No |
| | 7p. | Swollen lymph nodes? | ☐ Yes ☐ No |
| | 7q. | Menstrual cycle changes? | ☐ Yes ☐ No |
| | 7r. | Other/Comment? | ☐ Yes ☐ No<br>Specify: ___________________ |

© 2023 Elias MD et al. *JAMA Network Open*.

| | | | |
|---|---|---|---|
| **INSTRUCTIONS**: If no symptoms reported in question 7, skip to question 10. | | | |
| 8. | Did the participant call or see a medical provider due to side effects after vaccination? | | ☐ Yes *(go to 8a1)* ☐ No *(go to 9)* |
| | 8a1. | Outpatient phone call with primary care provider? | ☐ Yes ☐ No |
| | 8a2. | Outpatient visit with primary care provider? | ☐ Yes ☐ No |
| | 8a3. | Outpatient visit with specialty care provider (ex: cardiologist)? | ☐ Yes ☐ No |
| | 8a4. | Urgent care or Emergency Department Visit? | ☐ Yes ☐ No |
| | 8a5. | Hospital admission? | ☐ Yes ☐ No |
| | 8a6. | Hospital admission to intensive care unit? | ☐ Yes ☐ No |
| | 8b. | Were any tests performed as part of the evaluation? | ☐ Yes *(go to 8b1)* ☐ No *(go to 9)* |
| | 8b1. | Chest X-ray? | ☐ Yes ☐ No |
| | 8b2. | EKG? | ☐ Yes ☐ No |
| | 8b3. | Holter monitor, 7-14 day patch monitor, event monitor? | ☐ Yes ☐ No |
| | 8b4. | Troponin level? | ☐ Yes ☐ No |
| | 8b5. | Echocardiogram? | ☐ Yes ☐ No |
| | 8b6. | Cardiac MRI? | ☐ Yes ☐ No |
| | 8b7. | Other | ☐ Yes ☐ No<br>Specify: ____________________ |

© 2023 Elias MD et al. *JAMA Network Open*.

| | | | |
|---|---|---|---|
| 9. | Did the participant take medications for symptoms? | | ☐ Yes *(go to 9a1)* ☐ No *(go to 10)* |
| | 9a1. | Acetaminophen/Tylenol? | ☐ Yes ☐ No |
| | 9a2. | Ibuprofen/Motrin/Advil? | ☐ Yes ☐ No |
| | 9a3. | Antibiotics? | ☐ Yes ☐ No |
| | 9a4 | IVIG? | ☐ Yes ☐ No |
| | 9a5. | Steroids? | ☐ Yes ☐ No |
| | 9a6. | Colchicine? | ☐ Yes ☐ No |
| | 9a7. | Other immunomodulatory medication? | ☐ Yes ☐ No<br>Specify: |
| | 9a8. | Other? | ☐ Yes ☐ No<br>Specify: ____________________ |
| 10. | How many eligible individuals in the participants household (living in the same home) are vaccinated for COVID-19? | | ☐ = All<br>☐ = Some<br>☐ = None |

| | |
|---|---|
| Name of Person Completing Form: | ______________________________ |
| Signature of Person Completing Form: | ______________________________ |
| Date Form Completed (MM/DD/YYYY): | ______________________________ |

© 2023 Elias MD et al. *JAMA Network Open*.
