# Supplementary material for: Examination of Adverse Reactions After COVID-19 Vaccination Among Patients With a History of Multisystem Inflammatory Syndrome in Children
Source: JAMA Netw Open. 2023 Jan 3;6(1):e2248987. doi: 10.1001/jamanetworkopen.2022.48987 (PMC9857632; doi:10.1001/jamanetworkopen.2022.48987)
Supplement: Supplement 2. — Pediatric Heart Network MUSIC Study Investigators [file jamanetwopen-e2248987-s002.pdf]

Supplemental Online Content: Nonauthor Collaborators
*First name, last name, and suffix (if applicable) are required and will appear in PubMed.

*Group Name(s): Pediatric Heart Network MUSIC Study Investigators							
*First Name and Middle Initial(s)	*Last Name	*Suffix (eg, Jr, III)	Academic Degrees	Institution	Location (city, state/province, country)	Role or Contribution, eg, chair, principal investigator	Group (if more than 1 Group listed in the byline) and/or Subgroup (eg, Steering Committee)
Gail	Pearson			National Heart, Lung, and Blood Institute	Bethesda, MD		
Victoria	Pemberton			National Heart, Lung, and Blood Institute	Bethesda, MD		
D'Andrea	Egerson			National Heart, Lung, and Blood Institute	Bethesda, MD		
Lynn	Mahony			University of Texas Southwestern Medical Center	Dallas, TX	Protocol Chair	
Julie	Miller			Data Coordinating Center	Newton, MA	Primary investigator	
Kerri	Hayes			Data Coordinating Center	Newton, MA		
Allison	Crosby-Thompson			Data Coordinating Center	Newton, MA		
Ayesha	Amarnath			Data Coordinating Center	Newton, MA		
James	Ambrosoli			Data Coordinating Center	Newton, MA		
Cassandra	Artis			Data Coordinating Center	Newton, MA		
Kay	Rubio			Data Coordinating Center	Newton, MA		
Chitra	Kinhikar			Data Coordinating Center	Newton, MA		
Amanda	Marshall			Data Coordinating Center	Newton, MA		
Devine	Mbiydzenyuy			Data Coordinating Center	Newton, MA		
Valentina	Kazlova			Data Coordinating Center	Newton, MA		
Chenwei	Hu			Data Coordinating Center	Newton, MA		
Lozan	Eyob			Data Coordinating Center	Newton, MA		
Beverly	Slayton			Data Coordinating Center	Newton, MA		
Lauren	DiStefano			Data Coordinating Center	Newton, MA		
Jami	Honig			Data Coordinating Center	Newton, MA		
Tiffany	Bowie			Data Coordinating Center	Newton, MA		
Rob	Nero			Data Coordinating Center	Newton, MA		
Emily	Birmingham			Data Coordinating Center	Newton, MA		
Robin	Rowe			Data Coordinating Center	Newton, MA		
Melissa	Joyce			Data Coordinating Center	Newton, MA		
Cole	Gallagher			Data Coordinating Center	Newton, MA		
Djenawa	Bowman			Data Coordinating Center	Newton, MA		
Mo	Zhang			Data Coordinating Center	Newton, MA		
Alissa	Mooney			Data Coordinating Center	Newton, MA		
Andreea	Dragulescu			Hospital for Sick Children	Toronto, Ontario, Canada	Co-investigator	
Christopher	Lam			Hospital for Sick Children	Toronto, Ontario, Canada	Co-investigator	
Rae	Yeung			Hospital for Sick Children	Toronto, Ontario, Canada	Co-investigator	
Beth	Gamulka			Hospital for Sick Children	Toronto, Ontario, Canada	Co-investigator	
Jessica	Bainton			Hospital for Sick Children	Toronto, Ontario, Canada		
Martha	Rolland			Hospital for Sick Children	Toronto, Ontario, Canada		
Patti	Walter			Hospital for Sick Children	Toronto, Ontario, Canada		
Simran	Mahanta			Boston Children's Hospital	Boston, MA		
Thomas	Giorgio			Boston Children's Hospital	Boston, MA		
Numaira	Khan			Boston Children's Hospital	Boston, MA		
Annette	Baker			Boston Children's Hospital	Boston, MA		
Lisa Jean	Buckley			Boston Children's Hospital	Boston, MA		
Jessica	Jones			Boston Children's Hospital	Boston, MA		
LaTina	Watson			Boston Children's Hospital	Boston, MA		
Anna	Clarke			Boston Children's Hospital	Boston, MA		
Tor-Samuel-Aleer	Leek			Boston Children's Hospital	Boston, MA		
Gwendolyn	Orav			Boston Children's Hospital	Boston, MA		
Tonia	Morrison			Children's Hospital of Philadelphia	Philadelphia, PA		

Supplemental Online Content: Nonauthor Collaborators
*First name, last name, and suffix (if applicable) are required and will appear in PubMed.

***First Name and Middle Initial(s)**	***Last Name**	***Suffix (eg, Jr, III)**	Academic Degrees	Institution	Location (city, state/province, country)	Role or Contribution, eg, chair, principal investigator	Group (if more than 1 Group listed in the byline) and/or Subgroup (eg, Steering Committee)
Katherine	Lupton			Children’s Hospital of Philadelphia	Philadelphia, PA		
Donna	Sylvester			Children’s Hospital of Philadelphia	Philadelphia, PA		
Dana	Albizem			Children’s Hospital of Philadelphia	Philadelphia, PA		
Kristin	Lanzilotta			Children’s Hospital of Philadelphia	Philadelphia, PA		
Grace	Marks			Children’s Hospital of Philadelphia	Philadelphia, PA		
Madison	Johnson			Medical University of South Carolina	Charleston, SC		
Megan	Bickford			Medical University of South Carolina	Charleston, SC		
Linda	Lambert			Primary Children’s Hospital	Salt Lake City, UT		
Lilly	Fagatele			Primary Children’s Hospital	Salt Lake City, UT		
Andrea	Curless			Primary Children’s Hospital	Salt Lake City, UT		
Mark	Russell			University of Michigan Health System	Ann Arbor, MI	Primary investigator	
Tammy	Doman			University of Michigan Health System	Ann Arbor, MI		
Marisa	Almaguer			Cincinnati Children’s Hospital Medical Center	Cincinnati, OH		
Lauryn	Dugan			Cincinnati Children’s Hospital Medical Center	Cincinnati, OH		
Kathleen	Rathge			Cincinnati Children’s Hospital Medical Center	Cincinnati, OH		
Elizabeth	Seibert			Cincinnati Children’s Hospital Medical Center	Cincinnati, OH		
Mary	Stumpf			Riley Children's Hospital	Indianapolis, IN		
Jennifer	Howell			Riley Children's Hospital	Indianapolis, IN		
Jyoti	Patel			Riley Children's Hospital	Indianapolis, IN		
Gloria	Mitscher			Riley Children's Hospital	Indianapolis, IN		
Melissa	Burnett			Children's Healthcare of Atlanta	Atlanta, GA		
Kolby	Sanders-Lewis			Children's Healthcare of Atlanta	Atlanta, GA		
Brooke	Evans			Children's Healthcare of Atlanta	Atlanta, GA		
William T.	Mahle			Children's Healthcare of Atlanta	Atlanta, GA		
Lazaros	Kochilas			Children's Healthcare of Atlanta	Atlanta, GA		
Madison	Rudow			Children's Healthcare of Atlanta	Atlanta, GA		
Susie	Gentry			Children's Healthcare of Atlanta	Atlanta, GA		
Kristie	Le			Children's Healthcare of Atlanta	Atlanta, GA		
Caitlen	Taylor			Children's Healthcare of Atlanta	Atlanta, GA		
Lara	Shekerdemian			Texas Children’s Hospital	Houston, TX	Primary investigator	
Elias	Moussi			Texas Children’s Hospital	Houston, TX		
Sandra	Pena			Texas Children’s Hospital	Houston, TX		
Ricardo	Pignatelli			Texas Children’s Hospital	Houston, TX		
Olukayode	Garuba			Texas Children’s Hospital	Houston, TX		
Asela	Liu			Texas Children’s Hospital	Houston, TX		
Brett	Anderson			Morgan Stanley Children’s Hospital	New York, NY	Primary investigator	
Chantal	Sanchez			Morgan Stanley Children’s Hospital	New York, NY		
Karen	Martyrosyan			Morgan Stanley Children’s Hospital	New York, NY		
Jacqueline R.	Szmuszkovicz			Children’s Hospital Los Angeles	Los Angeles, CA	Primary investigator	
Andrew L.	Cheng			Children’s Hospital Los Angeles	Los Angeles, CA		
Sindhu	Mohandas			Children’s Hospital Los Angeles	Los Angeles, CA		
Jodie K.	Votava-Smith			Children’s Hospital Los Angeles	Los Angeles, CA		
Shuo	Wang			Children’s Hospital Los Angeles	Los Angeles, CA		
Pierre C.	Wong			Children’s Hospital Los Angeles	Los Angeles, CA		
Kavita	Sharma			University of Texas Southwestern Medical Center	Dallas, TX	Primary investigator	
Maria	Martinez			University of Texas Southwestern Medical Center	Dallas, TX		
Parvin	Mohazabnia			University of Texas Southwestern Medical Center	Dallas, TX		
Wendy	Rojas			University of Texas Southwestern Medical Center	Dallas, TX		

Supplemental Online Content: Nonauthor Collaborators
*First name, last name, and suffix (if applicable) are required and will appear in PubMed.

***First Name and Middle Initial(s)**	***Last Name**	***Suffix (eg, Jr, III)**	Academic Degrees	Institution	Location (city, state/province, country)	Role or Contribution, eg, chair, principal investigator	Group (if more than 1 Group listed in the byline) and/or Subgroup (eg, Steering Committee)
Joseph	Block			Children's Wisconsin, Medical College of Wisconsin	Milwaukee, WI	Primary investigator	
Regina	Cole			Children's Wisconsin, Medical College of Wisconsin	Milwaukee, WI		
Jennifer	Maldonado			Children's Wisconsin, Medical College of Wisconsin	Milwaukee, WI		
Shubhika	Srivastava			Nemours Children's Hospital	Wilmington, DE	Primary investigator	
Carol	Prospero			Nemours Children's Hospital	Wilmington, DE		
Varsha	Zadokar			Nemours Children's Hospital	Wilmington, DE		
Ed	Williams			Nemours Children's Hospital	Wilmington, DE		
Michael	Carr			Ann & Robert Lurie Children's Hospital	Chicago, IL	Primary investigator	
Kathleen	Van't Hof			Ann & Robert Lurie Children's Hospital	Chicago, IL		
Colleen	Sullivan			Ann & Robert Lurie Children's Hospital	Chicago, IL		
Michael A.	Portman			Seattle Children's Hospital	Seattle, WA	Primary investigator	
Hidemi	Kajimoto			Seattle Children's Hospital	Seattle, WA		
Deepthi G.	Nair			Seattle Children's Hospital	Seattle, WA		
Mikayla A.	Beckley			Seattle Children's Hospital	Seattle, WA		
Joan	Pancheri			Rady Children's Hospital	San Diego, CA		
Katheryn	Crane			Rady Children's Hospital	San Diego, CA		
Ashraf	Harahsheh			Children's National Hospital	Washington, DC	Co-investigator	
Charles	Berul			Children's National Hospital	Washington, DC	Co-investigator	
Laura	Olivieri			Children's National Hospital	Washington, DC	Co-investigator	
Mitchell	Haverty			Children's National Hospital	Washington, DC		
Alix	Fetch			Children's National Hospital	Washington, DC		
Christina	Schott			Children's National Hospital	Washington, DC		
Sarah	Litt			Children's National Hospital	Washington, DC		
Jennifer	Nelson			Children's Mercy Hospital	Kansas City, MO		
Michelle	Hite			Children's Hospital Colorado	Aurora, CA		
Isabel	Glassmeyer			Children's Hospital Colorado	Aurora, CA		
Todd	Nowlen			Phoenix Children's Hospital	Phoenix, AZ	Co-investigator	
Ashley	Herzberg			Phoenix Children's Hospital	Phoenix, AZ		
Samantha	Stack			Phoenix Children's Hospital	Phoenix, AZ		
Jade	Porche			Phoenix Children's Hospital	Phoenix, AZ		
Susan	Park			Phoenix Children's Hospital	Phoenix, AZ		
Amy	Johnson			Phoenix Children's Hospital	Phoenix, AZ		
Divya	Shakti			University of Mississippi	Jackson, MS	Primary investigator	
Aimee S.	Parnell			University of Mississippi	Jackson, MS		
Michael D.	Weiland			University of Mississippi	Jackson, MS		
Phenique	Parker			University of Mississippi	Jackson, MS		
Jeffrey A.	Kertis			University of Mississippi	Jackson, MS		
Charlotte V.	Hobbs			University of Mississippi	Jackson, MS		
Candace M.	Howard			University of Mississippi	Jackson, MS		
Shivraj	Savadkar			University of Mississippi	Jackson, MS		
Aren E.	Worrell			University of Mississippi	Jackson, MS		
Madelyn R.	Barr			University of Mississippi	Jackson, MS		
Heather	Williams			University of Mississippi	Jackson, MS		
David	Gordy			University of Mississippi	Jackson, MS		
Preeti	Vemula			University of Mississippi	Jackson, MS		
Zachary	White			University of Mississippi	Jackson, MS		
Jemylle Grace S.	Morato			University of Mississippi	Jackson, MS		
Sandra	Tyler			University of Mississippi	Jackson, MS		

Supplemental Online Content: Nonauthor Collaborators
*First name, last name, and suffix (if applicable) are required and will appear in PubMed.

***First Name and Middle Initial(s)**	***Last Name**	***Suffix (eg, Jr, III)**	Academic Degrees	Institution	Location (city, state/province, country)	Role or Contribution, eg, chair, principal investigator	Group (if more than 1 Group listed in the byline) and/or Subgroup (eg, Steering Committee)
Krissie	Hock			University of Alabama	Birmingham, AL		
Michelle	Sykes			Valley Children’s Healthcare and Hospital	Madera, CA	Primary investigator	
Carl	Owada			Valley Children’s Healthcare and Hospital	Madera, CA		
Mayra	Lemus Rangel			Valley Children’s Healthcare and Hospital	Madera, CA		
Isaura	Macias			Valley Children’s Healthcare and Hospital	Madera, CA		
Gautham	Singh			Children’s Hospital of Michigan	Detroit, MI	Primary investigator	
Sanjeev	Aggarwal			Children’s Hospital of Michigan	Detroit, MI	Primary investigator	
Charmaine	Williams-Farr			Children’s Hospital of Michigan	Detroit, MI		
Nancy	Sullivan			Children’s Hospital of Michigan	Detroit, MI		
Aiman	Almasnaah			Children’s Hospital of Michigan	Detroit, MI		
Vishnu	Undyala			Children’s Hospital of Michigan	Detroit, MI		
Brian	Hannah			Children’s Hospital of Michigan	Detroit, MI		
Mary	McCall			Children’s Hospital of Michigan	Detroit, MI		
Jacob	Strelow			Dell Medical Center	Austin, TX		
Rachel	Downey			Dell Medical Center	Austin, TX		
Ken	Shaffer			Dell Medical Center	Austin, TX		
Chesney	Castleberry			Dell Medical Center	Austin, TX		
Lisa	Pomeroy			Dell Medical Center	Austin, TX		
Rachel	Amsellem			Dell Medical Center	Austin, TX		
Olga	Shamailova			Cohen Children’s Hospital	New Hyde Park, NY		
Marla	Johnston			Children's Hospital New Orleans	New Orleans, LA		
Juan Carlos	Muniz			Nicklaus Children's	Miami, FL	Primary investigator	
Moya	Chang			Nicklaus Children's	Miami, FL		
Ryan	Shea			University of North Carolina	Chapel Hill, NC	Primary investigator	
Miriam	Davis			University of North Carolina	Chapel Hill, NC		
Maryanne	Chrisant			DiMaggio Children's Hospital	Hollywood, FL	Primary investigator	
Danielle	Katz			DiMaggio Children's Hospital	Hollywood, FL		
Norma	Barton			DiMaggio Children's Hospital	Hollywood, FL		
Doris	Alaby			DiMaggio Children's Hospital	Hollywood, FL		
Paulette	Smith			DiMaggio Children's Hospital	Hollywood, FL		
Kevin	Friedman					Echocardiography Core Laboratory	
Francesca	Sperotto					Echocardiography Core Laboratory	
Ed	Marcus					Echocardiography Core Laboratory	
Raquel	Ferreira					Echocardiography Core Laboratory	
Patrick	McGeoghegan					Echocardiography Core Laboratory	
Michael	Taylor					MRI Core Laboratory	
Joshua	Germann					MRI Core Laboratory	
Andrew	Powell					MRI Core Laboratory	
Andrew	Mackie					Acting Chair, Protocol Review Committee	
Jogarao	Gobburu					Protocol Review Committee	
Sally	Hunsberger					Protocol Review Committee	
Patrick	McQuillen					Protocol Review Committee	
Michael	Spaeder					Protocol Review Committee	
Dianne	Atkins					Data and Safety Monitoring Board	
Craig	Broberg					Data and Safety Monitoring Board	
David J.	Driscoll					Data and Safety Monitoring Board	
Frank	Evans					Data and Safety Monitoring Board Executive Secretary	
Sally A.	Hunsberger					Data and Safety Monitoring Board	

Supplemental Online Content: Nonauthor Collaborators
*First name, last name, and suffix (if applicable) are required and will appear in PubMed.

***First Name and Middle Initial(s)**	***Last Name**	***Suffix (eg, Jr, III)**	Academic Degrees	Institution	Location (city, state/province, country)	Role or Contribution, eg, chair, principal investigator	Group (if more than 1 Group listed in the byline) and/or Subgroup (eg, Steering Committee)
Liza-Marie	Johnson					Data and Safety Monitoring Board	
Thomas J.	Knight					Data and Safety Monitoring Board	
Paul	Lipkin					Data and Safety Monitoring Board	
J. Philip	Saul					Data and Safety Monitoring Board Chair	
