# Supplementary material for: Examination of Adverse Reactions After COVID-19 Vaccination Among Patients With a History of Multisystem Inflammatory Syndrome in Children
Source: JAMA Netw Open. 2023 Jan 3;6(1):e2248987. doi: 10.1001/jamanetworkopen.2022.48987 (PMC9857632; doi:10.1001/jamanetworkopen.2022.48987)
Supplement: Supplement 3. — Data Sharing Statement [file jamanetwopen-e2248987-s003.pdf]

Data Sharing Statement

Elias. Examination of Adverse Reactions After COVID-19 Vaccination Among Patients With a History of Multisystem Inflammatory Syndrome in Children. *JAMA Netw Open*. Published January 03, 2023. doi:10.1001/jamanetworkopen.2022.48987

Data
Data available: No
